# Elucidating Emergence and Transmission of Multidrug-Resistant Tuberculosis in Treatment Experienced Patients by Whole Genome Sequencing

**DOI:** 10.1371/journal.pone.0083012

**Published:** 2013-12-11

**Authors:** Taane G. Clark, Kim Mallard, Francesc Coll, Mark Preston, Samuel Assefa, David Harris, Sam Ogwang, Francis Mumbowa, Bruce Kirenga, Denise M. O’Sullivan, Alphonse Okwera, Kathleen D. Eisenach, Moses Joloba, Stephen D. Bentley, Jerrold J. Ellner, Julian Parkhill, Edward C. Jones-López, Ruth McNerney

**Affiliations:** 1 Faculty of Infectious and Tropical Diseases, London School of Hygiene & Tropical Medicine, London, United Kingdom; 2 Faculty of Epidemiology and Population Health, London School of Hygiene & Tropical Medicine, London, United Kingdom; 3 Wellcome Trust Sanger Institute, Hinxton, Cambridge, United Kingdom; 4 Joint Clinical Research Centre, Kampala, Uganda; 5 Department of Medical Microbiology, Makerere University College of Health Sciences, Kampala, Uganda; 6 Mulago Hospital Tuberculosis Clinic, Mulago Hospital, Kampala, Uganda; 7 Department of Pathology, University of Arkansas for Medical Sciences, Little Rock, Arkansas, United States of America; 8 Uganda-Case Western Reserve University Research Collaboration, Kampala, Uganda; 9 Section of Infectious Diseases, Department of Medicine, Boston Medical Center and Boston University School of Medicine, Boston, Massachusetts, United States of America; University of California, San Francisco, United States of America

## Abstract

**Background:**

Understanding the emergence and spread of multidrug-resistant tuberculosis (MDR-TB) is crucial for its control. MDR-TB in previously treated patients is generally attributed to the selection of drug resistant mutants during inadequate therapy rather than transmission of a resistant strain. Traditional genotyping methods are not sufficient to distinguish strains in populations with a high burden of tuberculosis and it has previously been difficult to assess the degree of transmission in these settings. We have used whole genome analysis to investigate *M. tuberculosis* strains isolated from treatment experienced patients with MDR-TB in Uganda over a period of four years.

**Methods and Findings:**

We used high throughput genome sequencing technology to investigate small polymorphisms and large deletions in 51 *Mycobacterium tuberculosis* samples from 41 treatment-experienced TB patients attending a TB referral and treatment clinic in Kampala. This was a convenience sample representing 69% of MDR-TB cases identified over the four year period. Low polymorphism was observed in longitudinal samples from individual patients (2-15 SNPs). Clusters of samples with less than 50 SNPs variation were examined. Three clusters comprising a total of 8 patients were found with almost identical genetic profiles, including mutations predictive for resistance to rifampicin and isoniazid, suggesting transmission of MDR-TB. Two patients with previous drug susceptible disease were found to have acquired MDR strains, one of which shared its genotype with an isolate from another patient in the cohort.

**Conclusions:**

Whole genome sequence analysis identified MDR-TB strains that were shared by more than one patient. The transmission of multidrug-resistant disease in this cohort of retreatment patients emphasises the importance of early detection and need for infection control. Consideration should be given to rapid testing for drug resistance in patients undergoing treatment to monitor the emergence of resistance and permit early intervention to avoid onward transmission.

## Introduction

Tuberculosis (TB) caused by *Mycobacterium tuberculosis* (Mtb) is a major global health problem, with an estimated 8.7 million new cases and 1.4 million deaths each year [1]. The World Health Organisation (WHO) and Stop TB Partnership have set the ambitious target of global "elimination" of TB as a public health problem by 2050 [2] but the emergence of strains that are resistant to anti-tuberculosis drugs threatens to disrupt efforts to control the disease [3]. Multidrug-resistant TB (MDR-TB), which accounts for in excess of 150,000 deaths *per annum*, is resistance to at least isoniazid and rifampicin, the two key first-line anti-tuberculosis drugs. WHO have recently reported the highest global levels of drug resistance ever documented with 3.4% of new TB patients and 19.8% of previously treated cases having MDR-TB [4]. Patients with MDR-TB require prolonged treatment of at least 18 months with a cocktail of expensive drugs of heightened toxicity. If not provided with appropriate therapy patients may remain infectious and a source of onward transmission. Standard first-line treatment regimens include isoniazid, rifampicin, ethambutol and pyrazinamide, empirically supplemented in some cases with streptomycin when drug resistance is suspected [5]. Second line TB drugs include the fluoroquinolones, injectable aminoglycosides and oral bacteriostatic agents such as cycloserine or ethionamide [6]. The primary mechanism for acquiring resistance in Mtb is the accumulation of point mutations (SNPs) in genes coding for drug targets or converting enzymes and drug resistant disease arises through selection of mutants during inadequate treatment [7]. Multidrug resistant disease in previously treated patients is generally attributed to sequential selection of drug resistant mutants during inadequate therapy, whereas for new patients transmission of a resistant strain is assumed [8,9]. However, recent reports of outbreaks of MDR-TB in TB and HIV treatment clinics suggest that transmission may be a greater factor in the global emergence of drug resistant disease than previously assumed [10].

The Mtb genome is characterised by low sequence diversity [11,12] and molecular typing techniques such as spoligotyping, variable number tandem repeats (MIRU-VNTR) and IS6110 restriction fragment length polymorphism (RFLP) have been used for epidemiological and evolutionary applications [13] but recent investigations of clinical isolates suggest strains with identical DNA fingerprinting patterns may harbour substantial genomic diversity [14-17]. Second generation high throughput sequencing technologies (e.g. Illumina HiSeq2000 [18]) mean it is now possible to perform whole genome sequencing of Mtb on a large scale [19,20]. In this study we applied whole genome sequencing to provide a better understanding of the emergence and acquisition of drug resistance in patients attending the Mulago Hospital National Tuberculosis and Leprosy Program (NTLP) treatment clinic in Kampala, Uganda.

## Methods

### Ethics Statement

The AIDS Research Sub-Committee of the Uganda National Council of Science and Technology and the Institutional Review Boards at the University of Medicine and Dentistry of New Jersey and the London School of Hygiene & Tropical Medicine approved the study. All patients provided written consent.

### Study population and patients

Uganda is one of 22 countries recognized as having a high burden of TB with an estimated 67,000 incident cases during 2011 [1]. MDR-TB is estimated at 1.4% in new cases (having received less than four weeks of therapy) and 12% in previously treated cases [1]. From July 2003 to April 2007, we conducted a cohort study of 439 previously treated pulmonary TB patients attending the NTLP treatment centre at Mulago Hospital in Kampala, an 85-bed inpatient facility that serves as the national referral centre and the largest TB treatment clinic in Kampala. The study was undertaken prior to the introduction of second line treatment for MDR-TB in Uganda. Data obtained has been presented elsewhere [5,21]. MDR-TB was found in 12.7% of retreatment cases and was the only common risk factor for death during follow-up for both HIV-infected and HIV uninfected patients. No association was observed between HIV positivity and MDR-TB [21]. During the study period fifty four patients had MDR-TB on enrolment and a further 5 were found to have MDR-TB following treatment. Mtb isolates obtained during the study were archived for future investigation. Not all isolates obtained were available for the study due to a lack of storage capacity in the isolating laboratory and samples were not available for 18 (30%) of patients identified as having MDR-TB during the study period. 

### Sample collection and drug susceptibility testing

We performed whole genome sequencing of a convenience sample of Mtb isolates (n=51) from 41 patients, including samples collected longitudinally from five patients (n=15) (Table 1). The patients had previously received treatment for TB and were presenting with a recurrence either as relapsed cases, treatment failures, or after defaulting treatment. On attending the Mulago Clinic all patients had received the standard WHO-recommended category II retreatment regimen composed of 2 months of streptomycin (S), rifampicin (R), isoniazid (H), ethambutol (E), and pyrazinamide (Z); 1 month of R,H,E and Z; and 5 months of R,H and E (2SRHEZ/1RHEZ/5RHE) [6]. Full treatment records for previous episodes of tuberculosis self-reported by patients were not available. Patients were selected because they were found by phenotypic susceptibility testing to have disease resistant to at least isoniazid and rifampicin, either at enrolment or following treatment.

**Table 1:** Data for 41 tuberculosis patients who contributed samples.

Patient	No. samples	Sample collection dates	Age	Gender	Paris code	HIV	*Previous TB episodes
A70011	6	Jul 03-Aug 04	36	F	66	+	1
A70012	1	May 05	27	F	50	-	2
A70067	2	Sep 03-Apr 04	30	M	235	+	1
A70086	1	Oct 03	na	na	na	na	na
A70088	1	Oct 05	48	F	50	-	3
A70136	3	Nov 03-Dec 04	32	M	87	-	3
A70144	2	Nov 03-Apr 04	24	F	598	+	1
A70170	1	Jan 04	39	F	na	+	2
A70196	1	Feb-04	24	F	198	-	4
A70250	1	Apr 04	29	F	80	+	2
A70260	1	Apr 04	19	F	66	+	1
A70280	1	May 04	33	F	80	+	1
A70329	1	Jul 04	26	F	584	-	1
A70376	1	Oct 05	27	M	72	-	1
A70387	1	Sept 04	48	F	404	-	1
A70416	1	Nov 04	na	na	na	na	na
A70428	1	Nov 04	45	M	137	-	4
A70441	1	Dec 04	43	F	36	-	1
A70448	1	Dec 04	28	M	na	-	1
A70451	1	May 05	25	M	45	-	1
A70458	1	Jan 05	42	M	389	+	5
A70480	1	Feb 05	50	M	580	-	1
A70490	1	Mar 05	30	F	24	+	5
A70501	1	Apr 05	49	M	580	-	3
A70547	1	Feb 06	20	F	72	-	3
A70555	1	Jul 05	30	F	273	-	3
A70582	1	Aug 05	23	M	30	-	1
A70596	1	Sep 05	20	M	6	-	3
A70620	1	Nov 05	42	M	7	-	3
A70645	1	Jan 05	30	M	1	-	2
A70655	1	Jan 06	24	F	66	-	2
A70657	1	Mar 06	42	M	na	-	2
A70659	1	Mar 06	29	M	43	-	1
A70661	1	Mar 06	47	M	na	-	6
A70730	1	Aug 06	28	F	30	-	2
A70757	1	Sept 06	27	F	568	+	4
A70762	1	Sep-06	29	F	132	+	1
A70763	2	Sep 06-Apr 07	29	F	52	-	2
A70769	1	Oct 06	30	F	na	-	2
A70780	1	Oct 06	26	M	76	na	2
A70785	1	Nov 06	30	F	397	-	2

^*^ Number of TB episodes prior to enrolling in the study, as self reported by the patient. na = not available

Parish code=reported home location

Mtb isolates obtained by liquid culture were subjected to drug-susceptibility tests for streptomycin, isoniazid, rifampicin, pyrazinamide and ofloxacin using BACTEC 460 or MGIT 960 testing systems [22] at critical concentrations of 1, 0.1, 1.0, 100 µg/mL and 2 mg/mL respectively. Ethambutol was tested at 2.5 ug/ml in BACTEC 460 and 5 ug/ml in the MGIT 960. Isolates that were resistant to isoniazid and rifampicin were further tested for resistance to second-line drugs. Capreomycin, kanamycin, ethionamide, and para-aminosalicylic acid were tested using the Middlebrook 7H10 agar proportion method at critical concentrations of 10, 5, 5, and 2 mg/mL, respectively. After identification Mtb isolates were subcultured and aliquots stored frozen at minus 80°C prior to shipping to the LSHTM where they were subcultured on Lowenstein Jensen slopes. DNA for sequencing was extracted using the Bilthoven RFLP protocol[23]. Mtb grown on LJ slopes was treated with lysozyme, sodium dodecyl sulphate, proteinase K, N-cetyl-N,N,N-trimethyl ammonium bromide (CTAB) and chloroform-isoamyl alcohol prior to precipitation with isopropanol. 

### Sequencing and genetic variant analysis

Samples were subjected to whole genome sequencing and spoligotyping, a widely used Mtb genotyping tool based on the presence or absence of short spacer sequences in a region of direct repeats within the Mtb genome [24,25]. DNA for sequencing was extracted using a standardised protocol [23]. Spoligotypes were inferred *in silico* using the *SpolPred* software [26] and determined by the Kamerbeek methodology [24]. Spoligotypes were assigned following the International Data Base (SpolDB4) recommendations [27]. All samples (n=51) underwent whole genome sequencing with 76-base paired end reads, using Illumina HiSeq2000 technology [18]. The data processing pipeline used has been described previously [28]. The raw sequence data were mapped uniquely to a corrected H37Rv reference genome [29,30] using *bwa* [31]. The mappings allowed SNPs and small indels to be called using SAMtools/BCFtools [32] Larger indels were identified using a consensus from paired end mapping distance or split read approaches (*Breakdancer* [33], CREST [34], *Pindel* [35] and *Delly* [36]), followed by an assembly-validated strategy using *Velvet* software [37]. Only those variants of high quality (at least Q30, equating to 1 error per 1000) and supported by bi-directional reads were retained. In addition, we excluded polymorphisms with two or more missing genotypes as well all variants in highly variable gene families (e.g. *PPE/PE* loci) and non-unique regions established by assessing the uniqueness of 54-mers across the genome. Variation density maps were generated using Circos software (www.circos.com).

First we catalogued the polymorphisms and identified variants including single nucleotide polymorphisms (SNPs), insertions and deletions (indels) and large deletions. Second, using this genomic variation we assessed the degree of population structure. Third, we focused on identifying the incremental variant changes across clustered strains and in drug resistance profiles within patients over time. Clusters of samples with less than 50 SNPs variation were examined. Finally we assessed degree of similarity between isolates to infer possible transmission of drug resistant disease. For some analysis we investigated known drug candidate regions (Table 2). 

**Table 2:** Candidate drug resistance and putative efflux pump genes investigated.

**Function**	**Genes**
**Drug resistance**	
Streptomycin	*rpsL, rrs, gidB*
Isoniazid	*katG, furA, ahpC, inhA, kasA, ndh, iniA, iniB, iniC, embB, fbpC, fabG1, nat, fadE24, efpA, ndh, Rv1592c, Rv1772, Rv2242, fabD, accD6, proA, efpA, fadE24*
Rifampicin	*rpoA, rpoB, rpoC, rpoD, embB*
Ethambutol	*Rv3126, manB, rmlD emb, embA, embB, embC, iniB, iniA, iniC, embR, Rv3124*
Pyrazinamide	*pncA*
Ofloxacin	*gyrA, gyrB, iniA, iniB, iniC, embR*
Ethionamide	*etaA*
Cycloserine	*iniC*
**Efflux pumps**	*Rv0194, emrB, Rv1250, Rv1272c, Rv1273c, Rv1634, stp, efpA, bacA, mmr, drrA, drrB, drrC*

Taken from http://www.tbdreamdb.com/

 A clustering dendrogram was constructed using R statistical software, using SNP and indel data [38]. To provide further phylogenetic analysis a best-scoring maximum likelihood tree was computed with RAxML (version 7.4.2) [39] using SNPdata. 

## Results

### Genomic variation

A total of 51 isolates collected from 41 patients were investigated (Table 1). All patients had been diagnosed with MDR-TB either at enrolment or following treatment, representing 69% of MDR-TB cases and 9.3% of patients enrolled in the cohort. Half of the patients came from Kampala District (50%), and the majority of the remainder from surrounding districts. Some patients reported living in the same parish (Table 2), but no two patients reported living in the same village (a collection of 50-70 households). Of the 38 patients for whom HIV status was known 11 (29%) were seropositive. In summary, sequencing yielded a median of 20.6 million 76 base-pair (bp) reads per sample. The reads mapped uniquely to more than 95% of the genome with in excess of 100-fold coverage (median 314) and 96% of the genome was covered at least 10-fold. Of 8269 putative SNPs 6857 (84.6%) were high quality and included in the analysis. Of these high quality SNPs, the majority (3667, 53.4%) were observed in single isolates. The majority were located in coding regions (median 71.5%, range 69.3 - 75.6%), and of those the majority lead to non-synonymous changes in amino acids (median 58.0%, range 54.5 - 60.6%). (See Table S1 for details) Identical non-synonymous SNP profiles were observed with pairs of samples isolated at the same point in time (n=2) and in general, low variation was seen in longitudinal samples from the same patient (Table 3).There was representation within drug resistance (DR) candidates (156) and putative efflux pump genes (41) (See Tables S2 and S3 for details). There was little evidence of mixed infection or cross contamination of samples (21 heterozygous genotypes, 1 per ~17000 SNP positions). However, it should be noted that isolates were sub-cultured at least twice prior to DNA extraction when selection of a dominant population may have occurred. The SNP density (average 2.4 per kb) tended to be greater in DR candidate genes (average 4.1 per kb), with two genes *pnc*A and *gid* having over 20 SNP per kb (Figure S1 and Table S4). 

**Table 3:** Incremental SNPs in longitudinal samples from the same patient and clustered strains.

Sample group	Patient	Date	SIT	Spoligo family	Drug INH	Drug RMP	Compared to	SNPs	
								All	DR
1	A70763-1	Sep-06	302	X1	R	S	H37Rv	689 (336)	10 (7)
	A70763-2	Apr-07	1721	X1	R	R	A70763-1	15 (11)	2 (2)
2	A70011-1	Jul-03	0	O	S	S	H37Rv	570 (306)	7(6)
	A70011-2	Jul-03	0	O	-	-	A70011-1	0 (0)	0 (0)
	A70011-3	Oct-03	0	O	-	-	A70011-1	2 (2)	0 (0)
	A70011-4	Oct-03	0	O	-	-	A70011-1	2 (0)	0 (0)
	A70011-5	Jul-04	4	LAM3/S	R	R	H37Rv	527 (334)	13 (11)
	A70011-6	Aug-04	4	LAM3/S	R	R	A70011-5	2 (2)	1 (1)
3	A70067-1	Sep-03	288	CAS2	S	S	H37Rv	1060 (539)	15 (9)
	A70067-2	Apr-04	2867	T2	R	R	H37Rv	475 (246)	11 (9)
4	A70136-1	Nov-03	26	CAS1_DELHI	S	S	H37Rv	1049 (544)	19 (14)
	A70136-2	Aug-04	26	CAS1_DELHI	R	S	A70136-1	2 (2)	1(1)
	A70136-3	Dec-04	26	CAS1_DELHI	R	R	A70136-1	2 (2)	1(1)
5	A70144-1	Nov-03	288	CAS2	R	R	H37Rv	1037 (533)	18 (13)
	A70144-2	Apr-04	288	CAS2	R	R	A70144-1	4 (3)	1(1)
6	A70086	Oct-03	2356	X1	R	R	H37Rv	988 (685)	12 (10)
	A70458	Jan-05	2356	X1	R	R	A70086	20 (15)	8 (8)
7	A70441	Dec-04	59	LAM11_ZWE	R	R	H37Rv	893 (650)	13 (10)
	A70547	Feb-06	59	LAM11_ZWE	R	R	A70411	14 (13)	5 (5)
	A70659	Mar-06	59	LAM11_ZWE	R	R	A70411	5 (5)	2 (2)
	A70582	Aug-06	59	LAM11_ZWE	R	R	A70411	5 (5)	2 (2)
8	**A70260**	Apr-04	4	LAM3/S	R	R	H37Rv	824 (599)	12 (10)
	**A70785**	Nov-06	125	LAM3	R	R	A70260	6 (6)	3 (3)
	**A70011-5**	Jul-04	4	LAM 3/S	R	R	A70260	4 (4)	2 (2)
	**A70011-6**	Aug-04	4	LAM 3/S	R	R	A70269	4 (4)	2 (2)
9	A70329	Jul-04	52	T2	R	R	H37Rv	793 (572)	14 (10)
	**A70376**	Oct-05	52	T2	R	R	A70329	21 (13)	5 (5)
	**A70730**	Aug-06	52	T2	R	R	A70329	20 (12)	5 (5)
10	A70448	Dec-04	-	-	R	R	H37RV	818 (585)	11 (9)
	A70762	Sep-06	-	-	R	R	A70448	32 (17)	9 (8)
11	**A70144-1**	Nov-03	288	CAS2	R	R	H37Rv	1037 (533)	18 (13)
	**A70144-2**	Apr-04	288	CAS2	R	R	A70144-1	4 (3)	1 (1)
	**A70769**	Oct-06	288	CAS2	R	R	A70144-1	21 (18)	5 (5)
	**A70780**	Oct-06	288	CAS2	R	R	A70144-1	15 (12)	5 (5)

Cluster = isolates < 50 SNP variation; Drugs: INH = Isoniazid , RIF= Rifampicin, R = resistant, S = susceptible; DR = drug resistant candidates (see Table S3 for list) SNP ( ) = non-synonymous changes, **BOLD** = high genome similarity and shared SNPs for MDR

**Figure 1:**
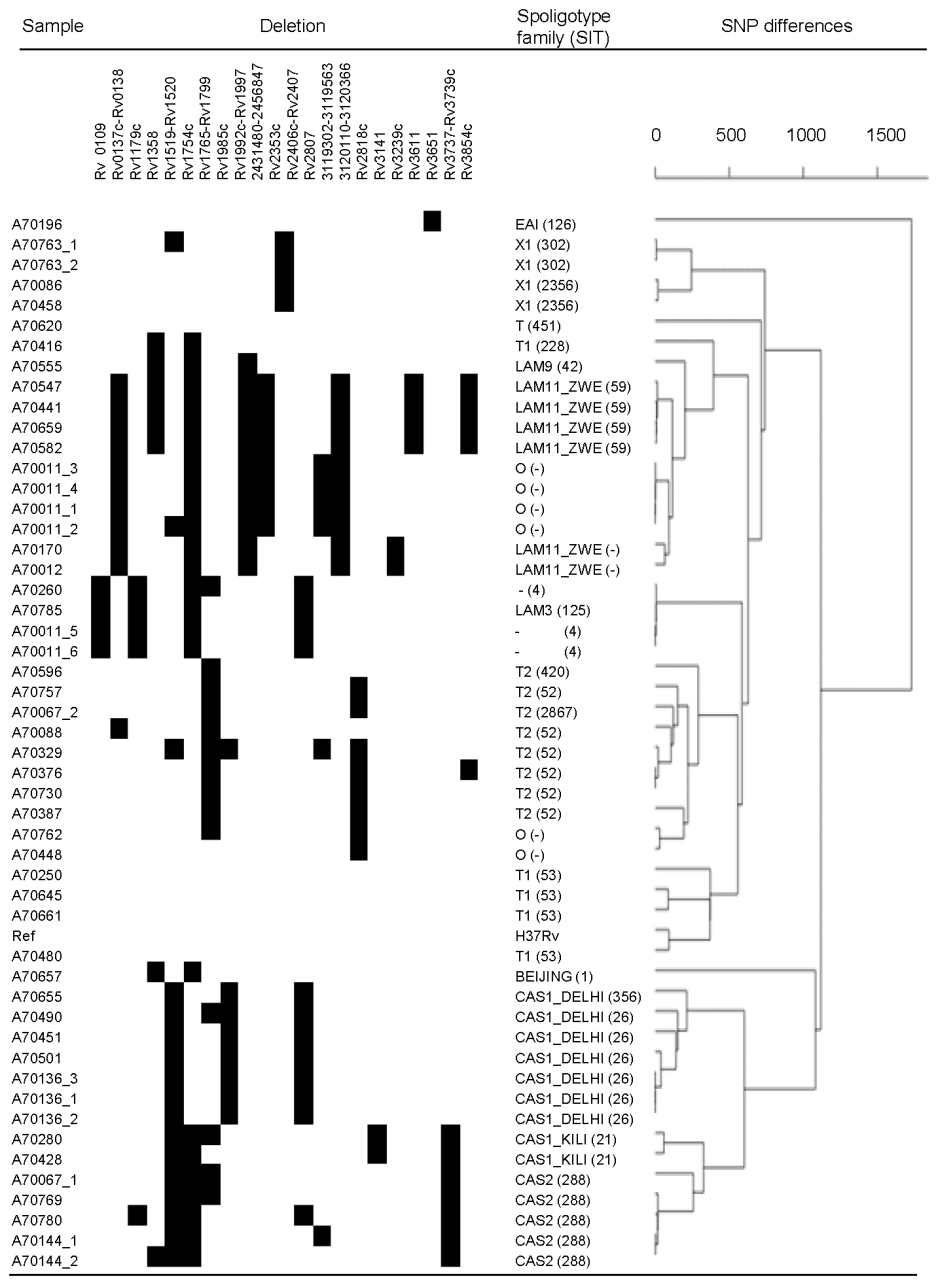
Population structure using SNP and small indel data with tabulated larger deletions. Clustering dendrogram constructed using R statistical software, based on a pair-wise identity. On average there are 860 SNP alleles and 64 small indels differences between any two isolates. Large deletions were identified using a consensus from paired end mapping distance or split read approaches followed by an assembly-validated strategy using Velvet software[37]. Only deletions considered informative are shown. SIT numbers were assigned in accordance in the international database SITVITWEB[41].

We observed 737 indels in unique and non-highly variable genetic regions, including 14 in DR genes. Of the 92 large deletions identified in robust regions of the genome 31 (33.7%) were detected in single isolates. The median number of deletions per isolate was 22 (range 13 - 27). Deletions considered informative are presented in Figure 1 and the full list is provided in Figure S2. All raw data can be downloaded (short read archive, accession number ERP000520) and a full list of variants can be found on the http://pathogenseq.lshtm.ac.uk/polytb website. 

### Population structure

The population structure of isolates based on SNP information separated samples into previously described lineages (Figure 1) [40]. Inclusion of larger variants did not change the clustering of samples which clustered to a first approximation by Spoligotype International Type (SIT), with EAI, LAM/T and Beijing/CAS ancestry separated [41]. Some divergance within isolates assigned by spoligotype to the T family was observed (samples A70620 and A70416). While over three hundred SNPs were informative for the CAS family (CAS1_DELHI, CAS2, CAS1_KILI) no informative SNP markers for the entire LAM family were identified, but there were strain-specific polymorphisms for LAM3&S convergent and LAM11_ZWE. Analysis of large deletions revealed putative markers for genotype families not previously reported, including SIT 59 and the larger LAM11_ZWE spoligotype family. 

With two exceptions, little variation was seen in isolates taken from the same patient over time (2-15 SNPs). For two patients (A70011 and A70067) longditudinal sampling indicated subsequent infection with a different strain of Mtb. (Table 3) In both cases the initial strain was drug sensitive and the second strain was MDR. A total of 37 (90%) patients had TB spoligotype patterns identical to those from one or more other patients. Of these 20 (54%) were found to be unique, having an excess of 50 SNPs variation and these stains were not implicated in transmission to other patients in the study. Six clusters (17 patients) with variation of less than 50 SNPs and the five sets of londitudinal samples were examined to ascertain the relatedness of the strains. Results are summarised in Table 3 and show a preponderance of SNPs in genes associated with drug resistance. In samples collected from the same patient over time there was a trend to increased numbers of SNPs. For clustered isolates orginating from different patients no correration was observed between incremental SNP and the date of sample collection (Spearman‘s correlation [42]).

**Figure 2:**
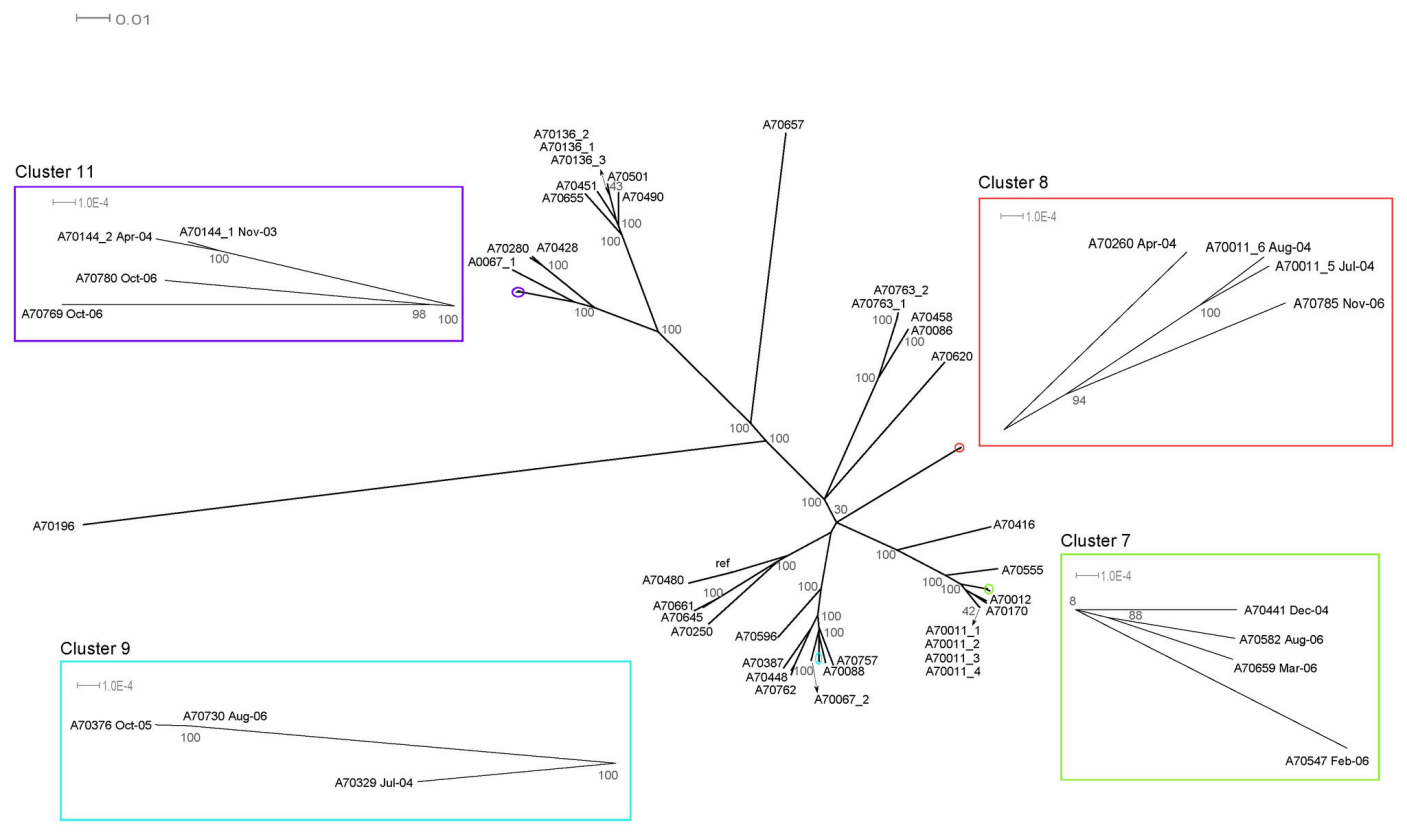
Radial phylogram constructed using SNPs and showing clustered samples. The best-scoring maximum likelihood phylogenetic tree was constructed using the set of 6,847 SNP sites. Support values computed from 100 bootstrap replicates provide assessment of confidence for each clade and are shown at the nodes of the tree. SNP variations within the clusters are summarised in Table 3.

Analysis of SNP differences found two paired samples from different patients (Cluster 6 and 10) with differing mutations predictive of MDR in *rpo*B and *kat*G (resistance to rifampicin and isoniazid respectively), suggesting resistance had emerged independently in these strains and excluding the possibility of transmission of MDR-TB (Table S5). The phylogeny of the remaining four clusters is presented in Figure 2. All isolates shared polymorphisms in *kat*G predictive of resistance to isoniazid (S315T) but there was variation in *rpo*B predictive of resistance to rifampicin. Review of base calls did not reveal evidence of subpopulations in these samples. In cluster 7 whereas the four isolates shared polymorphisms in *kat*G sample A700582 had discordant polymorphisms in *rpo*B suggesting independant emergence of MDR within this cluster. In cluster 9 there was strong evidence of transmission of MDR-TB, where two isolates (A70376 and A70730) differed by a single SNP and shared polymorphisms in *rpo*B and *kat*G. The third isolate (A70329) had differing polymorphism in *rpo*B and thus was not part of a common MDR transmission chain. Cluster 11 also provided evidence suggestive of transmision of MDR, with 4 isolates from 3 patients exhibiting identical polymorphims at two loci for both *rpo*B and *kat*G and a single *pncA* loci. Evidence of the continued acquisiton of polymorphisms was evident as the 2004 sample from patient A70144 exhibited a different mutation (S450Stop) from the two samples collected in 2006, (A70769 and A70780) which shared an additional rpoB mutation S450L. 

Cluster 8 included four isolates from three patients assigned SIT 4 (3) and SIT125 (1) by spoligotyping. The spoligotype pattern for sample A70785 lacked spacer 39, an observation seen both *in silico* analysis using *SpolPred* software and by the Kamerbeek methodology. Nineteen large deletions were common to all four isolates. Two additional deletions were observed in sample A70260 (Figure S2). SNPs in genes associated with MDR-TB (*rpo*B and *kat*G) were common across the 4 isolates and it is probable that these strains resulted from a common source of MDR-TB. 

**Figure 3:**
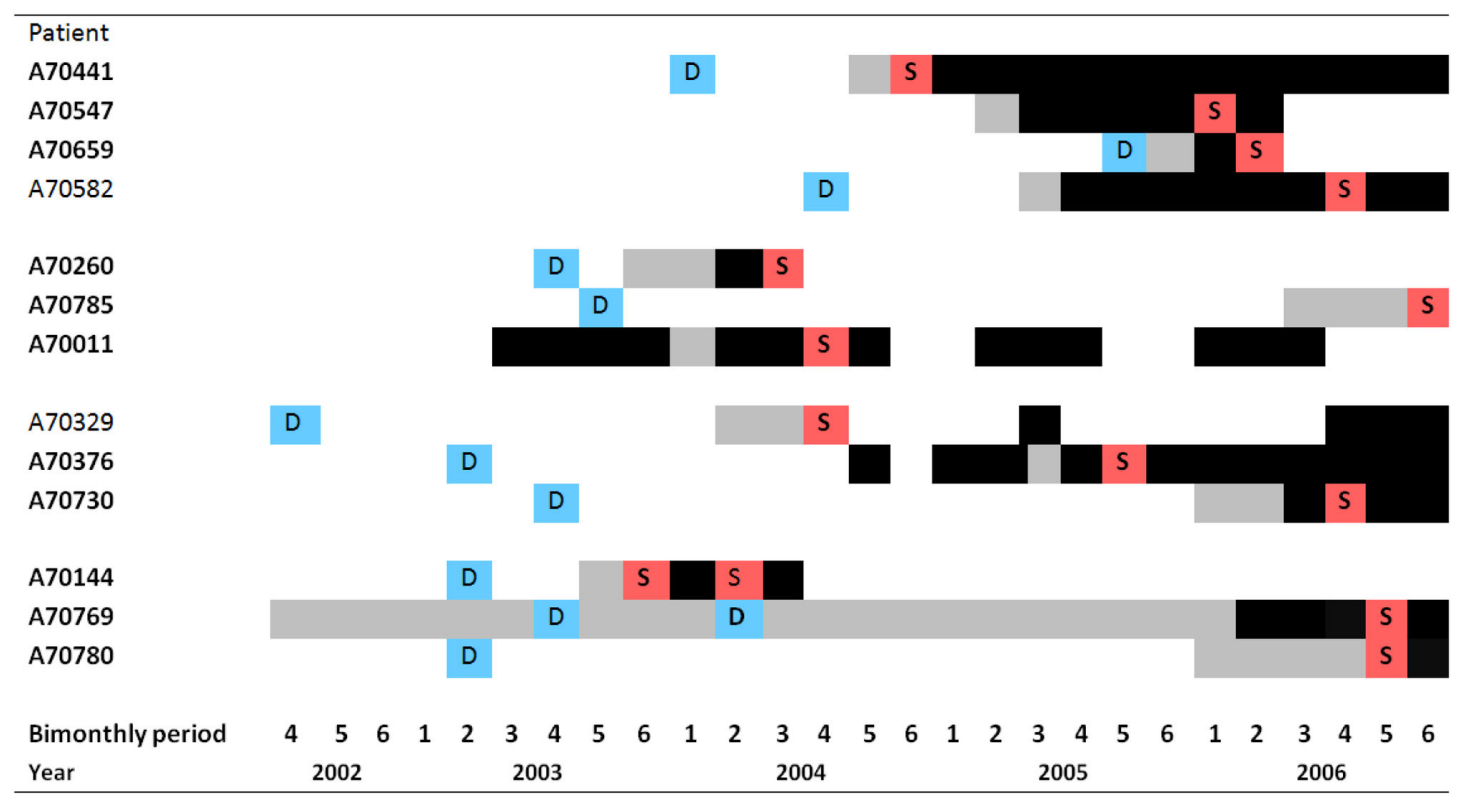
Episodes of tuberculosis for clustered patients. D = Date of diagnosis of initial TB episode as self reported by patient; S = date collection of sequenced sample; Black shading = Microbiologically proven TB; Grey shading = Duration of symptoms prior to diagnosis for episode when the sequenced sample was collected, as reported by patient; Bold = strain implicated in transmission of MDR.

Opportunities for transmission between clustered patients where transmission is suspected are not obvious as they resided in different neigbourhoods within the Kampala district. Examination of admission and discharge dates for patients admitted to the wards during the period of study suggest that clustered patients were not hospitalised concurrently (Table S6) Examination of cases histories and previous episodes of TB revealed concurrent episodes of disease raising the possibility of noscomial transmission during attendance at treatment clinics (Table 4 and Figure 3). 

**Table 4:** Mutations in *rpo*B in patient isolates and dates of TB episodes.

**Cluster No**	**Patient**	**rpoB codon (mutation)**	**Sample date**	**Enrollment**	**Symptoms weeks**	**Previous TB**	**Treatment end/death**
7	A70441	450 (TCG/TTG)	Dec-04	Dec-04	4	Jan-04	
7	A70547	450 (TCG/TTG)	Feb-06	Jun-05	8	Jun-00	Apr 06
7	A70659	450 (TCG/TTG)	March-06	Feb-06	20	Oct-05	
7	A70582	445 (CAC/CGC)	Aug-06	Aug-05	3	Aug-04	
**8**	**A70260**	**450 (TCG/TTG)**	**Apr-04**	**Apr-04**	**8**	**Aug-03**	**Jun-04**
**8**	**A70785**	**450 (TCG/TTG)**	**Nov-06**	**Nov-06**	**11**	**Oct-03**	
**8**	**A70011**	**450 (TCG/TTG)**	**Jul-04**	**Jul-03**	**20**	**Jul-03**	**Apr-05**
9	A70329	445 (CAC/CGC)	Jul-04	Jul-04	24	Jul-02	
**9**	**A70376**	**450 (TCG/TTG)**	**Oct-05**	**Sep 04**	**2**	**Mar-04**	**Apr-05**
**9**	**A70730**	**450 (TCG/TTG)**	**Aug-06**	**Aug 06**	**8**	**Jul-04**	**Mar 07**
**11**	**A70144**	**450 (TCG/TAG)**	**Nov-03**	**Nov 03**	**4**	**Apr 03**	
		**876 (GGT/A);1075 (GCT/C)**					
**11**	**A70769**	**450 (TCG/TTG)**	**Apr-04**	**Oct 06**	**208**	**Aug-03/Apr-05**	
		**876 (GGT/A);1075 (GCT/C)**					
**11**	**A70780**	**450 (TCG/TTG)**	**Oct-06**	**Oct 06**	**32**	**Feb-03/Sept-05**	
		**876 (GGT/A); 1075 (GCT/C)**					

Previous TB = date of previous TB diagnosis as self-reported by patient. Enrollment = Date of enrollment in study at Mulago TB Clinic. Symptoms = duration of symptoms prior to most recent diagnosis as self-reported by patient. **BOLD** = samples with high genome similarity and shared SNPs for MDR

### Drug resistance

Of the 51 isolates, 47 had phenotypic data on susceptibility to first line anti-tuberculosis drugs and 31 to second line drugs (Table 5). Isolates collected from three patients were reported phenotypically sensitive to all drugs tested and the remainder were resistant to at least two drugs. Forty two isolates were reported as MDR-TB, and 2 were resistant to isoniazid but not rifampicin In MDR-TB isolates the most common SNPs were *ka*tG S315T (28/42, 66.7%) and *rpo*B S531L (21/40, 52.5%), respectively (*rpo*B *E. coli* codon numbering). (Table S2 for details). Mutations not previously associated with drug resistance were identified in DR genes of strains found resistant. SNPs were observed in genes such as *rpo*C, suspected of compensatory properties [43-45]. In addition to the SNPs, 11 indels were observed in DR genes. Deletion analysis revealed five patient isolates lacked the gene *eth*A/*eta*A (Rv3854c) required for activation of ethionamide [46,47]. Similarly *katG*, required for catalase-peroxidase activation of isoniazid was deleted in two strains. Neither of the isolates deletions were implicated in transmission, possibly reflecting reduced fitness of these strains [48,49]. It should be noted that polymorphisms in DR genes were not observed in all samples found resistant by phenotypic testing. Three isoniazid resistant isolates contained no detectable mutation in the isoniazid candidate genes, two isolates had the R463L mutation in *kat*G, which is not associated with isoniazid resistance [50]. Three isolates resistant to rifampicin by phenotypic methods (liquid culture) contained no detectable mutation in *rpo*B. 

**Table 5:** Drug susceptibility of isolates ordered by genome population.

Sample	Spoligotype family	SIT no.	1st line drug susceptibility SIREP	2nd line drug susceptibility OCKPE
A70196	EA15	126	SRRSS	SSSSR
A70763_1	X1	302	RRSSS	-
A70763_2	X1	302	RRRSS	-
A70086	X1	2356	RRRRR	-SSSS
A70458	X1	2356	RRRRR	SSSSS
A70620	T	451	SRRRR	RSSSS
A70416	T1	228	SRRSS	SSSSS
A70555	LAM9	42	SRRRS	SSSSS
A70547	LAM11_ZWE	59	RRRRR	-
A70441	LAM11_ZWE	59	RRRSS	SSSSS
A70659	LAM11_ZWE	59	SRRRR	SSSSS
A70582	LAM11_ZWE	59	SRRSS	SSSSS
A70011_3	O	-	-	
A70011_4	O	-	-	
A70011_1	O	-	SSSSS	-SSSS
A70011_2	O	-	-	
A70170	LAM11_ZWE	1549	RRRRR	SSSSS
A70012	LAM11_ZWE	1549	SRRRS	S----
A70260	-	4	RRRRR	SSSSS
A70785	LAM3	125	RRRRR	SSSSS
A70011_5	-	4	RRRRR	SSSSS
A70011_6	-	4	RRRRR	-
A70596	T2	420	RRRRS	SSSSS
A70757	T2	52	RRRRR	S----
A70067_2	T2	2867	SRRRR	SSSSS
A70088	T2	52	SRRRR	S----
A70329	T2	52	RRRRS	SSSSR
A70376	T2	52	RRRRR	S----
A70730	T2	52	SRRRR	SSSSS
A70387	T2	52	SRRRS	SSSSS
A70762	O	-	RRRRS	S----
A70448	O	-	RRRRS	SSSSS
A70250	T1	53	SRRSR	SSSSS
A70645	T1	53	RRRRR	SSSSS
A70661	T1	53	RRRRR	SSSSS
A70480	T1	53	RRRSS	SSSSS
A70657	BEIJING	1	RRRRR	SSSSS
A70655	CAS1_DELHI	356	SRRRS	SSSSS
A70490	CAS1_DELHI	26	SRRRS	S----
A70451	CAS1_DELHI	26	RRRRS	S----
A70501	CAS1_DELHI	26	RRRRR	RSSSS
A70136_3	CAS1_DELHI	26	SRRRS	SSSSS
A70136_1	CAS1_DELHI	26	SSSSS	-
A70136_2	CAS1_DELHI	26	SRSRS	S----
A70280	CAS1_KILI	21	SRRRS	SSSSS
A70428	CAS1_KILI	21	RRRRS	SSSSS
A70067_1	CAS2	288	SSSSS	-
A70769	CAS2	288	RRRSS	SSSSS
A70780	CAS2	288	SRRRS	SSSSS
A70144_1	CAS2	288	SRRRS	SSSSS
A70144_2	CAS2	288	SRRRR	S----

First line drugs S: streptomycin; I: isoniazid; R: rifampicin; E: ethambutol; P: pyrazinamide

Second line drugs O: Ofloxacin; C: Capreomycin; K: Kanamycin; P: PAS (Para-Aminosalicylate Sodium); E: ethionamide

## Discussion

Our study has adopted a whole genome sequencing approach to investigate Mtb isolated from treatment experienced TB cases attending a clinic in Uganda and provides important insights into changes in within patient samples over time. Spoligotyping was shown to be a poor indicator for transmission of MDR-TB and we demonstrate the known advantage of SNPs as robust markers for population genetic analysis [17]. Samples were known to include strains resistant to multiple drugs and to encompass those strains with multiple polymorphisms in genes related to pharmacological action a high threshold (< 50 SNPs variation) was used to define clusters for the initial analysis. The low mutation rate that was observed is consistent with other reports [12,14,51,52]. No mixed infections were observed; however, a weakness of the study is that detection of mixed infections is hampered by the necessity to subculture isolates prior to extracting DNA for sequencing which may have altered the bacterial population [53]. In addition to SNPs, indels and large deletions were informative allowing us to differentiate patient isolates to a degree not previously accomplished. Using this approach we demonstrated that the majority of isolates from the cohort tested were not identical, ruling out direct transmission of MDR-TB between patients in these cases. However two patients were found to have acquired MDR-TB strains and isolates in three clusters (8 patients) were found to have highly similar genomes, suggesting that their disease was the result of transmission of MDR-TB. It should be noted that the isolates examined represented just 69% of patients diagnosed with MDR-TB and additional evidence transmission may have not been recorded due to sampling limitations. In some clusters distinct polymorphisms predictive of resistance were observed, suggesting resistance had emerged independently in these patients, all of whom were treatment experienced. That accumulation of polymorphisms in isolates from these patients was predominantly in genes related to drug function is not surprising and it would be expected that mutation rates would be influenced by the treatment experienced by individual patients. 

The genomic evidence of transmission of MDR-TB is supported by weaker circumstantial evidence of patient interaction and how, or when, transmission may have occurred is not apparent. The chronic nature the disease which may take months or years to emerge and the long delay in accessing care reported by some patients makes transmission events difficult to ascertain by traditional epidemiology. Opportunities for transmission were enhanced because patients with MDR-TB are likely to have remained infectious during treatment with ineffective standard therapies. 

We have also demonstrated genome sequencing as an efficient means of identifying putative markers of resistance. However, assignation of such markers will require validation using data from a larger collection of samples, including strains found susceptible to the drug by phenotypic testing methods. There are considerable challenges to overcome regarding the validation of such markers. As demonstrated in this study, and reported elsewhere, phenotypic methods of assessing drug susceptibility where the bacteria are grown in the presence of the drug may disagree with the presence or absence of polymorphisms at loci associated with resistance [54-57]. There is also a lack of knowledge regarding the clinical significance of SNPs as predictors of treatment effectiveness and studies to validate genomic markers require both high quality microbiological and clinical support [58]. 

The deletion of *eta*A reported to be required for activation of ethionamide [46] has not previously been reported and further work is required intreprete this finding as phenotypic test results available for 3 of the 5 isolates concerned suggest they were susceptible to the drug. The high density of SNPs in *pnc*A and *gid* genes might appear surprising compared to the overall stability of the genome. *PncA* encodes pyrazinamidase which is involved in the conversion of nicotinamide to nicotinic acid. It also hydrolyzes pyrazinamide to its active form pyrazinoic acid and numerous polymorphisms in this gene have been associated with resistance [59]. All patients had been exposed to this drug during treatment for previous episodes of TB. SNPs in *gid* have been reported to confer low-level streptomycin resistance in bacteria. In Mtb they have been observed to occur with high frequency, when they are associated with the emergence of high level resistance to streptomycin [60]. All patients in this study were exposed to this drug as part of their retreatment regimen, a strategy which has been shown to be unsatisfactory for patients with MDR-TB in this setting [5].

In conclusion we have demonstrated the utility of whole genome sequence analysis for investigating *M. tuberculosis* isolated from treatment exposed TB patients. That two patients acquired MDR strains during or following treatment for drug susceptible disease and a total of eight patients shared almost identical Mtb strains to those from one or more other patients, demonstrates that transmission may be an important source of MDR-TB in previously treated patients. Our data emphasises the importance of infection control to prevent transmission of drug resistant disease among patients receiving treatment, particularly in those settings where access to effective second line treatment remains limited. Early detection of MDR is more crucial than previously recognised in this setting and consideration should be given to implementing rapid tests for drug resistance as part of treatment monitoring. 

## Supporting Information

Figure S1 **Variation density map for 51 samples.**
(PDF)

Figure S2 **Large deletions detected.**
(PDF)

Table S1 **Sequencing data from 51 *M. tuberculosis* isolates.**
(PDF)

Table S2 **SNPs in drug resistance candidate genes and putative efflux pump genes.**
(PDF)

Table S3 **Frequency of previously reported and previously unreported (or not validated) non-synonymous polymorphisms in genes associated with drug resistance in isolates found resistant by phenotypic testing.**
(PDF)

Table S4 **Genes with SNP densities greater than 10 per kilobase.**
(PDF)

Table S5 **SNP in genes associated with drug resistance in clustered patient isolates.**
(PDF)

Table S6 **TB episodes, hospitalization dates and home location (village) of clustered patients.**
(PDF)
