# Supplementary material for: Elucidating Emergence and Transmission of Multidrug-Resistant Tuberculosis in Treatment Experienced Patients by Whole Genome Sequencing
Source: PLoS One. 2013 Dec 11;8(12):e83012. doi: 10.1371/journal.pone.0083012 (PMC3859632; doi:10.1371/journal.pone.0083012)

Figure S1 Variation density map for 51 samples.

Variation density maps for SNPs (blue) and small indels (orange) were generated using Circos software (www.circos.com). The dark grey concentric lines represent 1 variant/kb, the light grey lines represent 0.5 variant/kb.

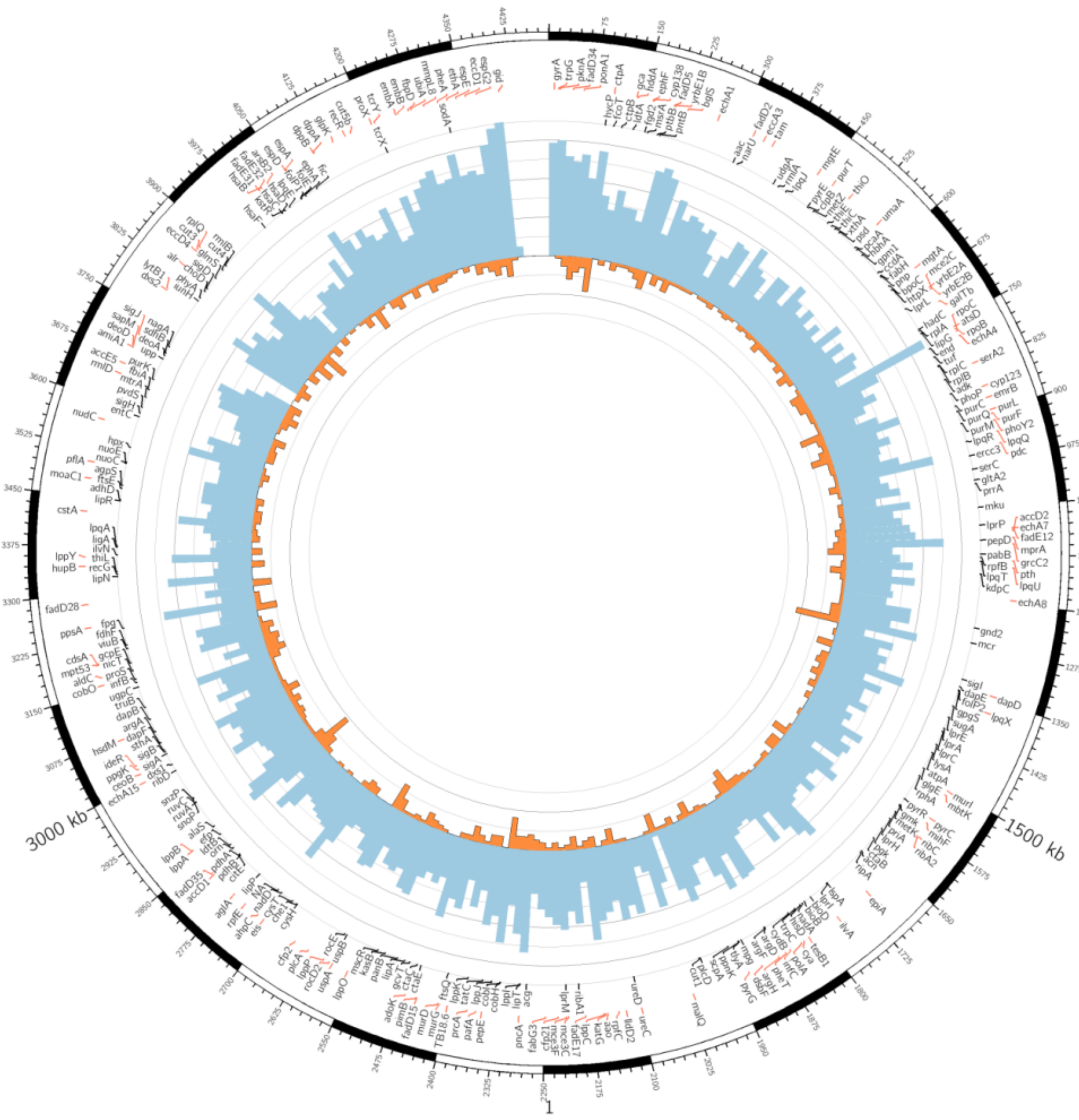

Supplement: Figure S1 — Variation density map for 51 samples. (PDF) [file pone.0083012.s001.pdf]
