# Supplementary material for: Elucidating Emergence and Transmission of Multidrug-Resistant Tuberculosis in Treatment Experienced Patients by Whole Genome Sequencing
Source: PLoS One. 2013 Dec 11;8(12):e83012. doi: 10.1371/journal.pone.0083012 (PMC3859632; doi:10.1371/journal.pone.0083012)

Figure S2   Large deletions detected.

92 Deletions were identified using a consensus of Breakdancer, CREST, Delly, and Pindel software calls. The deletions are represented in black, within a grid consisting of columns representing locations (genes), and the rows individual isolates (identifiers on the left, spoligotypes on the right). 92 large deletions were identified in robust regions of the genome. Median number per isolate 22, range 13 – 27; median size 1132bp, range 200bp – 30kb, Putative markers for genotype families not previously reported, include for SIT 4 (Rv109), SIT 59 (Rv3611) and for the LAM family (Rv1992c-Rv1997 and 2431480-2456847).

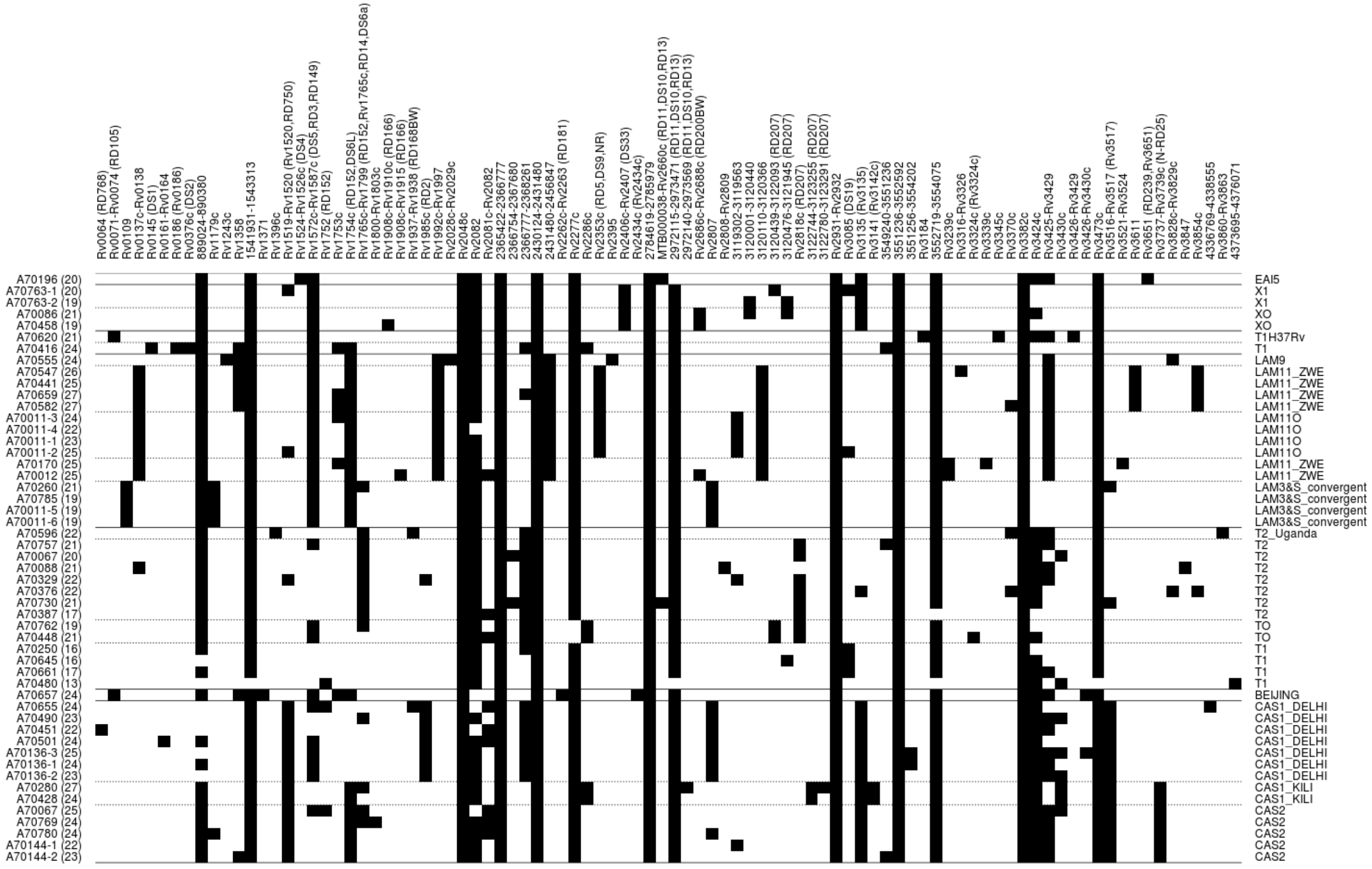

Supplement: Figure S2 — Large deletions detected. (PDF) [file pone.0083012.s002.pdf]
