# Supplementary material for: Elucidating Emergence and Transmission of Multidrug-Resistant Tuberculosis in Treatment Experienced Patients by Whole Genome Sequencing
Source: PLoS One. 2013 Dec 11;8(12):e83012. doi: 10.1371/journal.pone.0083012 (PMC3859632; doi:10.1371/journal.pone.0083012)
Supplement: Table S1 — Sequencing data from 51 M. tuberculosis isolates. (PDF) [file pone.0083012.s003.pdf]

Table S1 Sequencing data from 51 M. tuberculosis isolates.

Sample	*Reads*	*% reads mapped**	*Median Coverage*	*% genome >= 10-fold coverage*	***SNP***	***Non-cod***	***Coding***	***%NS***	***DR***	***DR (NS)***	***Small indels****	***Large del****
A70011-1	19193524	96.9	302	97.0	867	228	639	57.3	7	6	33	23
A70011-2	22640706	97.5	354	96.5	872	234	638	56.4	7	6	33	25
A70011-3	17001736	97.4	274	97.3	869	228	641	57.7	7	6	30	24
A70011-4	17974458	97	281	97.5	883	232	651	58.2	7	6	26	22
A70011-5	16930688	97.7	255	98.1	826	227	599	55.1	13	11	35	19
A70011-6	23346066	97.9	373	97.6	824	226	598	54.5	14	12	18	19
A70012	17012534	97.5	244	96.4	876	236	640	56.7	9	7	45	25
A70067-1	18208838	95.7	253	97.9	1480	447	1033	58	15	9	72	25
A70067-2	26983446	98	452	97.4	797	214	583	58	11	9	32	20
A70086	19078890	98	309	98.0	988	303	685	58.1	12	10	37	21
A70088	25147636	97.5	399	98.4	803	226	577	59.6	12	8	15	21
A70136-1	14492390	96.8	210	97.7	1436	429	1007	59.3	19	14	88	24
A70136-2	21398140	96.8	320	97.8	1434	426	1008	58.5	20	15	38	23
A70136-3	24655036	96.8	332	97.7	1439	428	1011	58.9	20	15	44	25
A70144-1	16875312	97.2	254	98.1	1470	450	1020	58	18	13	71	22
A70144-2	24633680	97.3	380	98.0	1481	453	1028	57.6	19	14	50	23
A70170	18077412	98	293	97.2	877	229	648	56.3	11	8	34	25

A70196	15400876	97.8	239	97.2	2111	603	1508	59.7	27	17	112	20
A70250	17820996	97.8	274	97.9	588	171	417	58.5	9	4	26	16
A70260	23936824	97.9	379	97.2	824	225	599	54.6	12	10	41	21
A70280	21806080	97.7	348	97.9	1458	445	1013	58.3	19	14	47	27
A70329	21774006	97.3	339	98.2	793	221	572	60.1	14	10	29	22
A70376	30641304	97.6	499	98.6	809	224	585	60	15	11	8	22
A70387	27376032	97.6	431	98.0	828	236	592	59.5	12	11	22	17
A70416	21644984	97.7	345	97.2	894	240	654	55.7	13	11	51	24
A70428	18276516	95.9	292	97.5	1440	434	1006	57.9	15	10	64	24
A70441	24210068	97.8	407	96.4	893	243	650	55.8	13	10	44	25
A70448	20917066	97.4	345	97.3	818	233	585	59.1	11	9	43	21
A70451	14669382	97.4	231	97.7	1440	431	1009	59.4	20	15	92	22
A70458	17807802	98.1	290	97.7	1002	306	696	58.2	12	10	35	19
A70480	17683274	97.3	264	98.4	316	77	239	55.2	3	2	25	13
A70490	20854946	96.1	295	97.7	1432	429	1003	58.8	18	13	45	23
A70501	20577712	97.4	332	97.2	1435	432	1003	58.4	19	13	58	24
A70547	9718984	96.5	151	97.4	888	235	653	56.4	12	9	63	26
A70555	10580428	97	161	96.8	877	239	638	56.4	13	10	67	24
A70582	21693876	97.4	345	97.2	873	231	642	57	10	7	20	27
A70596	26221094	97.5	419	98.3	784	224	560	58.8	9	7	11	22

A70620	12980070	97.7	202	98.5	979	282	697	55.7	8	6	78	21
A70645	7355440	96.9	112	97.9	553	165	388	60.6	7	6	45	16
A70655	29793910	97.2	464	98.5	1452	427	1025	58.9	20	14	24	24
A70657	18095606	97.3	298	97.4	1453	436	1017	59.3	14	9	47	24
A70659	33646664	97.8	541	97.7	889	238	651	56.8	12	9	6	27
A70661	17959800	97.2	291	98.1	578	172	406	58.9	6	5	18	17
A70730	17251132	97.6	281	98.1	801	223	578	59.9	15	11	22	21
A70757	20118936	96.9	314	98.3	807	218	589	59.1	11	7	15	21
A70762	19730884	96.9	299	98.3	834	235	599	59.6	13	11	23	19
A70763-1	21549334	97.8	342	98.2	1016	303	713	57.4	8	5	23	20
A70763-2	21536794	97.6	341	98.1	1015	302	713	57.9	9	6	26	19
A70769	25718434	96.6	406	98.5	1489	447	1042	57.8	19	14	30	24
A70780	30107214	97.9	477	98.8	1513	446	1067	57.5	19	14	24	24
A70785	23819592	97.8	382	98.4	830	224	606	55.8	12	10	22	19

Reads = number of reads, * reads mapped uniquely. SNP = number of SNPs at an error rate of 1 per 1000; Non-cod = number of non-coding SNPs, Coding = number of coding SNPs, %NS = percentage of non-synonymous coding SNPs, Indels = number of small insertions and deletions; Large del = number large deletions; * = after quality control.
