# Supplementary material for: Elucidating Emergence and Transmission of Multidrug-Resistant Tuberculosis in Treatment Experienced Patients by Whole Genome Sequencing
Source: PLoS One. 2013 Dec 11;8(12):e83012. doi: 10.1371/journal.pone.0083012 (PMC3859632; doi:10.1371/journal.pone.0083012)
Supplement: Table S2 — SNPs in drug resistance candidate genes and putative efflux pump genes. (PDF) [file pone.0083012.s004.pdf]

Table S2 SNPs in drug resistance candidate genes and putative efflux pump genes

Gene	Start	End	Size	# snps	Dens. /kb	Mutations (position, allele & amino acid changes, codon, # samples)
pncA	2288689	2289249	561	15	26.7	2288712 (C->A,V->I,Ns,179,1); 2288725 (C->T,M->I,Ns,174,1); 2288726 (A->T,M->M,=,174,1); 2288765 (C->T,G->V,Ns,161,0.5); 2288783 (A->T,L->L,=,155,0.5); 2288928 (C->A,G->R,Ns,107,2); 2288981 (A->G,I->S,Ns,89,1); 2289006 (G->C,H->H,=,81,1); 2289038 (T->C,H->P,Ns,70,0.5); 2289051 (A->G,S->A,Ns,66,1); 2289055 (G->A,S->S,S,64,7); 2289062 (T->C,D->A,Ns,62,1); 2289200 (C->T,G->V,Ns,16,0.5); 2289215 (T->G,D->G,Ns,11,2); 2289239 (A->G,L->W,Ns,3,1);
gid	4407533	4408207	675	15	22.2	4407593 (T->C,A->A,S,204,16); 4407763 (T->G,S->G,Ns,148,1); 4407810 (G->C,A->A,=,132,1); 4407878 (C->A,V->V,S,109,1); 4407907 (G->A,L->I,Ns,100,1); 4407932 (T->G,E->E,S,91,1); 4407966 (A->G,I->R,Ns,80,1); 4407985 (G->C,P->P,=,74,1); 4408053 (C->T,C->F,Ns,51,1); 4408062 (A->G,L->R,Ns,48,1); 4408077 (A->C,L->P,Ns,43,1); 4408099 (C->T,G->*,Ns,36,1); 4408120 (C->G,G->G,=,29,3); 4408130 (C->G,L->L,=,25,1); 4408161 (A->C,L->P,Ns,15,12);
rpsL	781564	781938	375	3	8.0	781680 (C->T,T->T,S,39,1); 781691 (A->G,K->R,Ns,43,2); 781826 (A->G,K->R,Ns,88,3);
ethA	4326010	4327479	1470	9	6.1	4326042 (A->G,F->V,Ns,479,1); 4326236 (T->C,D->A,Ns,414,5); 4326285 (A->G,S->A,Ns,398,2); 4326356 (G->A,S->Y,Ns,374,1); 4326411 (C->A,D->N,Ns,356,1); 4327073 (A->C,L->P,Ns,135,1); 4327113 (G->A,H->N,Ns,122,2); 4327153 (C->T,W->C,Ns,108,3); 4327273 (C->T,W->C,Ns,68,1);
embB	4246520	4249816	3297	16	4.9	4246910 (G->A,V->M,Ns,131,4); 4246958 (C->T,R->C,Ns,147,1); 4247112 (C->A,P->H,Ns,198,0.5); 4247435 (A->GT,M->VL,NsNs,306,7); 4247437 (G->ACT,M->III,NsNsNs,306,17); 4247475 (A->C,Y->S,Ns,319,1); 4247580 (A->C,D->A,Ns,354,2); 4247652 (A->C,E->A,Ns,378,1); 4247735 (G->A,G->S,Ns,406,0.5); 4247736 (G->AC,G->DA,NsNs,406,5.5); 4248009 (A->G,Q->R,Ns,497,1); 4248010 (G->CT,Q->HH,NsNs,497,2); 4248031 (A->C,E->D,Ns,504,1); 4248447 (C->T,T->I,Ns,643,1); 4249414 (G->A,P->P,S,965,4); 4249589 (G->A,D->N,Ns,1024,1);
rpoB	759811	763329	3519	17	4.8	760318 (G->T,V->F,Ns,170,1); 760979 (T->C,M->T,Ns,390,1); 761006 (C->T,T->I,Ns,399,1); 761080 (T->G,F->V,Ns,424,1); 761104 (C->A,Q->K,Ns,432,2); 761113 (G->T,D->Y,Ns,435,1); 761114 (A->GT,D->GV,NsNs,435,3.5); 761143 (C->AGT,H->NDY,NsNsNs,445,9); 761144 (A->G,H->R,Ns,445,2); 761159 (C->AGT,S->*WL,NsNsNs,450,24.5); 762105 (C->T,R->R,S,765,1); 762280 (C->A,R->S,Ns,824,1); 762289 (C->T,R->C,Ns,827,1); 762315 (C->A,H->Q,Ns,835,1); 762438 (T->G,G->G,S,876,14); 762718 (G->A,V->M,Ns,970,0.5); 763035 (T->C,A->A,S,1075,16);
Rv3124	3489513	3490382	870	4	4.6	3489672 (C->T,P->S,Ns,54,14); 3489858 (G->T,A->S,Ns,116,1); 3489911 (G->A,L->L,S,133,4); 3490248 (C->T,L->F,Ns,246,2);
katG	2153896	2156118	2223	10	4.5	2154231 (C->G,G->G,=,629,1); 2154545 (T->G,Q->R,Ns,524,1); 2154731 (C->A,R->Q,Ns,462,16); 2154746 (A->T,L->L,=,457,1); 2154841 (G->C,Y->Y,=,425,1); 2155175 (C->GT,S->SI,=Ns,314,33); 2155302 (C->A,G->S,Ns,272,1); 2155548 (A->C,W->R,Ns,190,1); 2155564 (G->A,F->L,Ns,184,1); 2155826 (T->C,Y->S,Ns,97,1);

gyrA	7302	9818	2517	11	4.4	7539 (A->G,T->A,Ns,80,10); 7572 (T->C,S->P,Ns,91,1); 7585 (G->C,S->T,Ns,95,47); 8040 (G->A,G->S,Ns,247,1); 8438 (C->T,Y->Y,S,379,1); 8452 (C->T,A->V,Ns,384,1); 8513 (C->G,V->V,S,404,1); 8688 (G->T,A->S,Ns,463,1); 9143 (T->C,I->I,S,614,1); 9276 (C->T,L->L,S,659,2); 9304 (G->A,G->D,Ns,668,47);
emrB	876822	878444	1623	6	3.7	876861 (C->T,Q->H,Ns,527,1); 876985 (G->C,P->P,=,486,1); 877228 (C->A,G->D,Ns,405,4); 877787 (T->G,R->G,Ns,219,2); 878178 (G->A,L->L,S,88,4); 878325 (C->T,G->G,S,39,1);
Rv1634	1839175	1840590	1416	5	3.5	1839267 (T->G,L->L,S,31,1); 1839313 (A->G,I->V,Ns,47,1); 1839336 (G->A,R->R,S,54,1); 1839766 (G->C,G->R,Ns,198,16); 1840061 (T->C,V->A,Ns,296,2);
ahpC	2726200	2726787	588	2	3.4	2726294 (G->A,G->D,Ns,32,3); 2726679 (G->A,E->E,S,160,1);
Rv0194	226878	230462	3585	12	3.3	227098 (T->C,M->T,Ns,74,50); 227252 (A->G,Q->Q,S,125,4); 227707 (C->T,A->V,Ns,277,1); 228069 (G->A,V->M,Ns,398,1); 228168 (G->C,G->R,Ns,431,11); 228333 (C->A,L->M,Ns,486,2); 228960 (C->T,Q->*,*,695,1); 228990 (T->A,F->I,Ns,705,1); 229172 (C->T,A->A,S,765,1); 229435 (C->T,P->L,Ns,853,2); 230170 (C->T,P->L,Ns,1098,1); 230195 (C->G,A->A,S,1106,1);
rmlD	3646902	3647816	915	3	3.3	3647048 (A->G,S->A,Ns,256,1); 3647379 (C->T,A->A,S,145,1); 3647598 (A->G,N->K,Ns,72,1);
Rv1772	2006643	2006954	312	1	3.2	2006763 (C->T,R->W,Ns,41,4);
embA	4243239	4246523	3285	10	3.0	4243466 (C->T,C->C,S,76,1); 4243628 (G->A,T->T,S,130,2); 4243698 (G->A,G->S,Ns,154,4); 4243715 (C->T,A->A,S,159,4); 4243854 (G->A,V->M,Ns,206,1); 4244063 (C->A,G->G,S,275,1); 4245061 (C->A,T->N,Ns,608,9); 4245172 (C->A,P->Q,Ns,645,1); 4245889 (G->A,G->D,Ns,884,7); 4245975 (C->T,P->S,Ns,913,1);
embC	4239869	4243153	3285	9	2.7	4240677 (C->T,T->I,Ns,270,1); 4240783 (G->A,E->E,S,305,1); 4241048 (A->G,N->D,Ns,394,1); 4241298 (T->G,L->R,Ns,477,0.5); 4241568 (G->A,R->H,Ns,567,2); 4242081 (G->A,R->Q,Ns,738,14); 4242188 (G->T,A->S,Ns,774,1); 4242649 (C->T,R->R,S,927,50); 4242809 (G->C,V->L,Ns,981,4);
Rv2242	2515312	2516556	1245	3	2.4	2515333 (C->T,P->S,Ns,8,7); 2516279 (T->C,M->T,Ns,323,14); 2516344 (C->T,P->S,Ns,345,1);
Rv1273c	1422309	1424057	1749	4	2.3	1422673 (C->T,G->V,Ns,461,7); 1422674 (C->T,G->W,Ns,461,7); 1422927 (T->C,T->T,S,376,1); 1423631 (A->C,C->R,Ns,142,1);
drrA	3272221	3273216	996	2	2.0	3273114 (C->A,A->A,S,298,14); 3273145 (C->G,H->D,Ns,309,1);
gyrB	5240	7267	2028	4	2.0	6112 (G->C,M->I,Ns,291,1); 6124 (C->T,A->A,S,295,1); 6140 (G->T,V->L,Ns,301,11); 6501 (G->A,R->H,Ns,421,1);
fbpC	156578	157600	1023	2	2.0	157129 (C->T,G->C,Ns,157,14); 157292 (C->T,E->D,Ns,102,12);
stp	2606715	2608328	1614	3	1.9	2607722 (T->C,I->L,Ns,202,1); 2608124 (C->A,D->N,Ns,68,21); 2608263 (G->A,D->E,Ns,21,1);
nat	4007337	4007954	618	1	1.6	4007570 (C->T,G->*,Ns,128,4);
proA	2724237	2725484	1248	2	1.6	2724936 (G->A,A->A,S,182,1); 2725479 (G->A,T->T,S,1,1);
kasA	2518123	2519373	1251	2	1.6	2518140 (C->T,T->T,S,6,1); 2518927 (G->A,G->S,Ns,269,1);
bacA	2062816	2064735	1920	3	1.6	2062929 (T->C,I->L,Ns,602,1); 2063692 (C->T,L->F,Ns,347,1); 2063918 (A->G,I->S,Ns,272,1);
iniA	410839	412761	1923	3	1.6	411372 (T->C,G->G,S,178,4); 412018 (C->G,Q->E,Ns,394,10); 412281 (T->G,H->Q,Ns,481,1);

ndh	2101658	2103049	1392	2	1.4	2102140 (G->A,Q->K,Ns,303,0.5); 2102997 (A->G,V->G,Ns,17,4);
iniB	409363	410802	1440	2	1.4	410265 (C->T,A->A,S,301,1); 410281 (T->G,L->V,Ns,307,2);
inhA	1674209	1675018	810	1	1.2	1674472 (C->T,L->L,S,88,6);
drrC	3274079	3274909	831	1	1.2	3274552 (G->A,L->L,S,158,1);
drrB	3273213	3274082	870	1	1.1	3273971 (G->A,K->K,S,253,4);
Rv1250	1394186	1395925	1740	2	1.1	1395017 (A->G,R->G,Ns,278,1); 1395562 (C->G,F->L,Ns,459,1);
fabD	2516795	2517703	909	1	1.1	2517618 (G->A,S->N,Ns,275,1);
manB	3644905	3645984	1080	1	0.9	3645531 (C->T,D->Y,Ns,151,14);
fadE24	3505370	3506776	1407	1	0.7	3506657 (A->C,I->L,Ns,430,7);
accD6	2520751	2522172	1422	1	0.7	2521436 (A->G,D->G,Ns,229,1);
iniC	412758	414239	1482	1	0.7	413262 (C->G,P->A,Ns,169,4);
efpA	3153046	3154638	1593	1	0.6	3154600 (G->A,N->K,Ns,12,2);
Rv1272c	1420417	1422312	1896	1	0.5	1420470 (G->A,H->N,Ns,613,1);
