# Supplementary material for: Elucidating Emergence and Transmission of Multidrug-Resistant Tuberculosis in Treatment Experienced Patients by Whole Genome Sequencing
Source: PLoS One. 2013 Dec 11;8(12):e83012. doi: 10.1371/journal.pone.0083012 (PMC3859632; doi:10.1371/journal.pone.0083012)
Supplement: Table S3 — Frequency of previously reported and previously unreported (or not validated) non-synonymous polymorphisms in genes associated with drug resistance in isolates found resistant by phenotypic testing. (PDF) [file pone.0083012.s005.pdf]

Table S3 Frequency of previously reported and previously unreported (or not validated) non-synonymous polymorphisms in genes associated with drug resistance in isolates found resistant by phenotypic testing.

Drug	Gene	Previously reported Mutation (No. isolates)	Putative Mutations (No. isolates)
Isoniazid	*kat*G	S315T (28), S315N (2), W191G (1)	A144T (2), Q525P (1), L458H (1), W191G (1), Y426STOP (1), G630R (1), Y98C (1)
	*ini*B		L307V (1)
	*ini*A		Q394E (9), H481Q (1)
	ndh		V18A (3), A304STOP (1)
Rifampicin	*rpo*B*	S531L (21), S531STOP (1), H526D (3), H526R (1), H526Y (2), H526N (1), D516G (1), D516V (3), D516Y (1), Q513K (2), F505V (1)	V251F (1), M471T (1), T480I (1), R905S (1), R908C (1), H916Q (1), V1051M (1)
Streptomycin	*rps*L	K88R (3), K43R (2)	
	rrs		c524t (2) (nucleotide position)
	gid	G30R (2)	L26F (1), L101F (1), I81T (1), E92D (1), S149R (1)
Pyrazinamide	*pnc*A	G17D (1), D12A (2), L4S (1), D63G (1), G162D (1), S67P (1).	G108STOP (2), H82D (1) V180F (1), L156Q (1), M175K (1)
Ethambutol	*emb*B	M306I (14), M306V (6), M306L (1), G406D (4), G406S (1), Q497R (1)	V131M (3), Q497H (2), T6431 (1), E594D (1), Y319S (1), D354A (1), D1024N (1)
	*emb*A		T608N (9), G884D (5), G154S (4).
	*emb*C		R567H (2)
Ofloxacin	*gyr*A	S91P (1)	A463S (1)
Ethionamide	*eth*A/*eta*A		F479V(1), D414A(5), S398A(2), S374Y(1), D356N(1), L135P(1), H122N(2), W108C(3), W68C(1)

*E.coli codon numbering
