# Supplementary material for: Elucidating Emergence and Transmission of Multidrug-Resistant Tuberculosis in Treatment Experienced Patients by Whole Genome Sequencing
Source: PLoS One. 2013 Dec 11;8(12):e83012. doi: 10.1371/journal.pone.0083012 (PMC3859632; doi:10.1371/journal.pone.0083012)
Supplement: Table S4 — Genes with SNP densities greater than 10 per kilobase. (PDF) [file pone.0083012.s006.pdf]

Table S4   Genes with SNP densities greater than 10 per kilobase.

In addition to pncA and gid, two other genes were found to have SNP densities greater than 20, a hypothetical protein Rv0095c and *esx*O, which encodes a secretary protein of the Esat-6 family believed to be of importance for virulence and growth.

Gene	***Size***	***Numbers SNPs***	***Density/kb***
Rv0095c	411	13	31.63
pncA	561	15	26.73
esxO	285	7	24.56
gid	675	15	22.22
Rv0277A	258	5	19.38
esxL	285	5	17.54
lppB	663	11	16.59
ubiA	909	13	14.30
TB7.3	216	3	13.89
Rv2561	294	4	13.61
esxD	324	4	12.35
Rv0061	426	5	11.74
mazE1	174	2	11.49
Rv2628	363	4	11.02
esxN	285	3	10.53
vapC6	384	4	10.42

Distribution of all densities: Min. 0.3178; 1st Qu. 1.2720; Median 1.2720; Mean 2.3920; 3rd Qu. 2.9630; Max. 31.6
