# Supplementary material for: Elucidating Emergence and Transmission of Multidrug-Resistant Tuberculosis in Treatment Experienced Patients by Whole Genome Sequencing
Source: PLoS One. 2013 Dec 11;8(12):e83012. doi: 10.1371/journal.pone.0083012 (PMC3859632; doi:10.1371/journal.pone.0083012)
Supplement: Table S5 — SNP in genes associated with drug resistance in clustered patient isolates. (PDF) [file pone.0083012.s007.pdf]

Table S5 SNP in genes associated with drug resistance in clustered patient isolates

Cluster 6		410281 iniB 307	761143 rpoB 445	761159 rpoB 450	781691 rpsL	2155175 katG 315	2289200 pncA	2289239 pncA	4242809 embC 981	4247437 embB	4248010 embB	4249414 embB	4407966 gid 80	Spoligotype
**Ref**		T	C	C	A	C	C	A	A	G	G	G	A	
**A70086**	Oct-03	**G**	C	**T**	**G**	**G**	CT	A	**C**	**A**	G	**A**	A	717776777760771
**A70458**	Jan-05	**G**	T	C	A	C	C	G	**C**	**A**	**T**	**A**	**G**	717776777760771

Cluster 7		6140 gyrB 301	761143 rpoB 445	761159 rpoB 450	1472760 rrs	2155175 katG 315	2289062 pncA 62	4247736 embB406	4248009 embB497	4248447 embB 643	4249589 embB1024	4408062 gid 48	4408161 gid 15	
**Ref**		G	C	C	A	C	T	G	A	C	G	A	A	
**A70441**	Dec-04	**T**	C	**T**	**C**	**G**	T	**A**	A	C	G	**G**	**C**	777777606060771
**A70547**	Feb-06	**T**	C	**T**	**C**	**G**	**C**	G	A	**T**	G	A	**C**	777777606060771
**A70659**	Mar-06	**T**	C	**T**	**C**	**G**	T	G	**G**	C	**A**	A	**C**	777777606060771
**A70582**	Aug-06	**T**	**G**	C	**C**	**G**	T	G	A	C	G	T	**C**	777777606060771

**Cluster 8**	**Date**	**7585 gyrA 95**	**9304 gyrA 178**	**411372 iniA 178**	**761159 rpoB 450**	**2102997 ndh 18**	**2155175 katG 315**	**4242649 embC 927**	**4243698 embA154**	**4246910 embB**	
**Ref**		G	G	T	C	A	C	C	G	G	
**A70785**	Nov-06	C	A	C	T	G	G	T	A	A	000000007760731
**A70260**	Apr-04	C	A	C	T	G	G	T	A	A	000000007760771
**A70011_5**	Jul-04	C	A	C	T	G	G	T	A	A	000000007760771
**A70011_6**	Aug-04	C	A	C	T	G	G	T	A	A	000000007760771

		762718 rpoB 970	764671 rpoC 433	766207 rpoC 945	767127 rpoC 1252	781826 rpsL 88	2288928 pncA 107	4247437 embB 306	4247580 embB 354	4407763 gid 148	4407907 gid 100	
**Ref**		G	G	G	G	A	C	G	A	T	G	
**A70785**	Nov-06	G	G	G	**T**	A	C	**A**	A	**G**	G	000000007760731
**A70260**	Apr-04	G	G	**T**	G	A	C	G	**C**	T	**A**	000000007760771
**A70011_5**	Jul-04	G	**AG**	G	G	**G**	**A**	**A**	A	T	G	000000007760771
**A70011_6**	Aug-04	**AG**	G	G	G	**G**	**A**	**A**	A	T	G	000000007760771

**Cluster 9**		**7539 gyrA 80**	**761143 rpoB 445**	**761159 rpoB 450**	**1674472 inhA 88**	**2155175 katG 315**	**2289215 pncA**	**2726294 aphC 32**	**4243715 embA 159**	**4245061 embA 601**	**4247437 embB**	**4247736 embB406**	**4327153 ethA 108**	**4408120 gid 29**	
**Ref**		A	C	C	C	C	T	G	C	C	G	G	C	C	
**A70329**	Jul-04	**G**	**G**	C	**T**	**G**	T	**A**	**T**	**A**	**A**	G	**T**	**G**	777777777760731
**A70376**	Oct-05	**G**	C	**T**	**T**	**G**	**G**	**A**	**T**	**A**	G	**A**	**T**	**G**	777777777760731
**A70730**	Aug-06	**G**	C	**T**	**T**	**G**	**G**	**A**	**T**	**A**	G	**A**	**T**	**G**	777777777760731

Cluster 10		7585 gyrA 95	9276 gyrA 659	412018 iniA 394	761104 rpoB 432	761143 rpoB 445	762315 rpoB 835	2155175 katG 314	2155548 katG 190	4245061 embA 608	4247435 embB	4248010 embB	4327113 ethA 122	
**Ref**		G	C	C	C	C	C	C	A	C	A	G	G	
**A70448**	Dec-04	**C**	**T**	**G**	C	**G**	C	**T**	A	A	**G**	G	**A**	777777403760731
**A70762**	Sep-06	**C**	**T**	**G**	**A**	C	**A**	**C**	**C**	A	A	**C**	**A**	777777403760731

Cluster 11		761159 rpoB	762438 rpoB 876	763035 rpoB 1075	2155175 katG 315	2289055 pncA	4242081 embC 738	4247435 embB	4247437 embB	4247736 embB	4326356 ethA 374	4327073 ethA 135	4407593 gid 204	
**Ref**		C	T	T	C	G	G	A	C	G	G	**A**	T	
**A70144-1**	Nov-03	**C**	**G**	**C**	**G**	**A**	**A**	A	C	G	G	**A**	**C**	700367700003771
**A70144-2**	Apr-04	**A**	**G**	**C**	**G**	**A**	**A**	A	C	G	G	**A**	**C**	700367700003771
**A70769**	Oct-06	**T**	**G**	**C**	**G**	**A**	**A**	A	**G**	**C**	**A**	**A**	**C**	700367700003771
**A70780**	Oct-06	**T**	**G**	**C**	**G**	**A**	**A**	**G**	**G**	G	G	**C**	**C**	700367700003771
