# Supplementary material for: Elucidating Emergence and Transmission of Multidrug-Resistant Tuberculosis in Treatment Experienced Patients by Whole Genome Sequencing
Source: PLoS One. 2013 Dec 11;8(12):e83012. doi: 10.1371/journal.pone.0083012 (PMC3859632; doi:10.1371/journal.pone.0083012)
Supplement: Table S6 — TB episodes, hospitalization dates and home location (village) of clustered patients. (PDF) [file pone.0083012.s008.pdf]

Table S6 TB episodes, hospitalization dates and home location (village) of clustered patients.

Cluster	Sample	Sample Date	Duration symptoms	Duration sampling	Admission	Discharge	Previous TB	Death	Village
**7**	A70441	Dec-04	4	Dec 04-Sep 06			Jan-04		224
**7**	A70547	Feb-06	8	Jun 05-Feb 06	23-Jun-05	7-Sep-05	Jun-00	01-Apr-06	167
**7**	A70659	Mar-06	20	Mar 06			Oct-05		43
**8**	A70260	Apr-04	8	Apr 04-Jun-04	28-Apr-04	23-Jun-04	Aug-03	27-Jun-04	405
**8**	A70785	Nov-06	11	Nov 06			Oct-03		397
**8**	A70011_5	Jul-04	5	Jul 03-Aug 04	10-Jul-03<br>27-Aug-04	29-Aug -03<br>25-Oct-04			404
**8**	A70011_6	Aug-04	5	Jul 03-Aug 04	10-Jul-03<br>27-Aug-04	29-Aug -03<br>25-Oct-04	Jul-03		404
**9**	A70376	Oct-05	2	Sep 04-Feb 06	1-Sep-04	5-Nov-04	Mar-04		444
**9**	A70730	Aug-06	8	Aug 06-Mar 07	3-Aug-06	11-Aug-06	Jul-04<br>Oct-04	11-Mar-07	188
**11**	A70769	Oct-06	208	Oct 06			Aug-03<br>Apr-05		-
**11**	A70780	Oct-06	32	Oct 06			Feb-03<br>Sept-05		503
**11**	A70144_1	Nov-03	4	Nov 03-Apr 04	21-Nov-03	16-Jan-04	Apr-03	1-Jul-04	-
**11**	A70144_2	Apr-04	4	Nov 03-Apr 04	21-Nov-03<br>01-Mar-04	16-Jan-03<br>05-Mar-04	Apr-03	1-Jul-04	-
